# [Retracted] All-*trans*-retinoic acid inhibits chondrogenesis of rat embryo hindlimb bud mesenchymal cells by down­regulating p53 expression

**DOI:** 10.3892/mmr.2026.13867

**Published:** 2026-03-30

**Authors:** Tao-Gen Zhang, Xue-Dong Li, Guo-Yong Yu, Peng Xie, Yun-Guo Wang, Zhao-Yong Liu, Quan Hong, De-Zhong Liu, Shi-Xin Du

Mol Med Rep 12: 210–218, 2015; DOI: 10.3892/mmr.2015.3423

Following the publication of the above paper, a concerned reader has drawn the Editor's attention to the fact that the western blot data used to portray the Sox9 and Col2a1 data in Fig. 1C were strikingly similar, although the dimensions of the bands in these figure parts differed. After having conducted an independent investigation of the data in this paper in the Editorial Office, it also came to light that various of the western blot protein bands featured in Figs. 1E, 3C and 4C bore striking similarities to data in other figure parts, where different experiments were intended to have been portrayed, albeit with the apparent resizing of the data bands in some cases, or in one case, where the experimental lanes in the figure parts were labelled differently (Fig. 1E vs. Fig. 3C).

Given the identification of these duplications of the data in this paper, the Editor of *Molecular Medicine Reports* has determined that it should be retracted from the Journal on account of a lack of confidence in the authenticity of the presented data. The authors were asked for an explanation to account for these concerns, but the Editorial Office did not receive a reply. The Editor regrets any inconvenience that has been caused to the readership of the Journal.

